# Automatic Oral Cancer Detection Using Improved Honey Badger Algorithm-Based Feature Selection

**DOI:** 10.3390/diagnostics16131969

**Published:** 2026-06-24

**Authors:** Nebras Sobahi, Yagmur Olmez, Osman Fatih Koparır, Muammer Turkoglu, Adalet Çelebi, Yazyd Alghamedi, Abdulkadir Şengür

**Affiliations:** 1 Department of Electrical and Computer Engineering, Faculty of Engineering, King Abdulaziz University, P.O. Box 80204, Jeddah 21589, Saudi Arabia; yalghamedi0003@stu.kau.edu.sa; 2 Department of Electrical and Electronics Engineering, Faculty of Engineering and Natural Sciences, Malatya Turgut Ozal University, 44210 Malatya, Türkiye; yagmur.olmez@ozal.edu.tr; 3 Department of Electrical-Electronics Engineering, Faculty of Technology, Firat University, 23100 Elazig, Türkiye; ksengur@firat.edu.tr; 4 Department of Histology and Embryology, Faculty of Veterinary Medicine, Fırat University, 23119 Elazig, Türkiye; 241304208@firat.edu.tr; 5 Department of Software Engineering, Faculty of Engineering and Natural Sciences, Samsun University, 55000 Samsun, Türkiye; muammer.turkoglu@samsun.edu.tr; 6 Oral and Maxillofacial Surgery Department, Faculty of Dentistry, Mersin University, 33000 Mersin, Türkiye; adaletcelebi@mersin.edu.tr

**Keywords:** oral cancer, feature selection, honey badger algorithm, histopathologic image

## Abstract

**Background/Objectives:** Oral cancer is one of the most common types of cancer, with high mortality rates if not detected early. Traditional diagnostic methods based on clinical examination rely on experience, leading to delays in early and reliable diagnosis. In recent years, medical imaging and AI-based computer-aided diagnostic systems have shown promising results in the automated identification of oral cancer. In particular, the efficient management of high-dimensional feature spaces in machine learning and deep learning approaches directly impacts classification performance. In this context, metaheuristic-based feature selection technics is a critical component because of eliminating redundant and irrelevant features. To address these challenges, this study proposes a metaheuristic-based feature selection method to reduce feature dimensionality and enhance the classification performance of oral cancer detection. **Methods:** This study proposes an improved Honey Badger Algorithm-based feature selection approach for the automated detection of oral cancer. In the proposed method, the distance vector used in the HBA method has been redefined to improve the balance between exploration and exploitation. Additionally, a new Cauchy mutation-based migration strategy was integrated into the proposed method to increase diversity in the search space and avoid getting stuck in local minima. The continuous-valued iHBA method was discretized with a modified sin–cos transfer function for feature selection. Oral cancer images were filtered using the CLAHE method, and after extracting deep features with the ResNet50 architecture, the proposed metaheuristic-based method was used to select discriminative features. **Results:** The proposed method was first tested for reliability and limitations through repeated runs on problems with different characteristics, such as unimodal and multimodal classical test functions. Then, the method was applied to extract significant features for oral cancer detection using a Histopathological Imaging Database containing 1224 histopathological oral tissue images at 100× and 400× magnification levels from 230 patients. The proposed approach was assessed in terms of accuracy, precision, recall, F1-score, and convergence curves in comparison with various classical feature selection techniques, such as wrapper-based, filter-based, and embedded-based methods, as well as other metaheuristic-based methods. The experimental results demonstrated that the suggested strategy outperformed both traditional feature selection techniques and alternative metaheuristic approaches. **Conclusions:** The effectiveness of the proposed method in improving diagnostic accuracy was evaluated through comprehensive experimental analyses. The obtained findings show that the proposed iHBA-based feature selection approach can reduce feature dimensionality, eliminate redundant and irrelevant features, and improve the classification performance of oral cancer detection. Therefore, the proposed method provides an effective and competitive computer-aided diagnostic framework for the automated classification of histopathological oral cancer images.

## 1. Introduction

In medical imaging, artificial intelligence (AI) has emerged as a promising tool for supporting earlier, faster, and more accurate disease diagnosis. The growing amount and complexity of medical images generated in clinical practice makes the manual examination of imaging data time intensive and specialist knowledge-dependent. AI-enabled systems can scan complex visual patterns, identify locations that seem suspect and help clinicians arrive at more consistent and educated conclusions on diagnosis [1]. Hence, it is expected that the application of AI in medical imaging will increase the efficiency of diagnosis, reduce the clinical strain and enable quicker patient care [2]. Therefore, recent studies have focused on deep learning-based approaches for automatic, reliable classification of medical images. In the study conducted by Soladoye et al., a dataset comprising 10,661 images obtained from 118 patients was used for the early detection of Acute Lymphoblastic Leukemia [3]. The transfer learning models based on VGG-19 and EfficientNet-B3 allowed early and reliable detection of illness. The findings showed that the accuracy of EfficientNet-B3 was 96%, which was better than VGG-19. Gülmez reviewed the current state and evolution of artificial intelligence (AI) and deep learning applications for colorectal cancer detection in recent years [4]. In this review, 110 high-quality publications and nine publicly available medical imaging datasets were comprehensively analyzed. The review covered deep learning architectures, including ResNet, the most commonly used architecture, as well as VGG, EfficientNet, Xception, Inception, DenseNet, Vision Transformer (ViT), Swin Transformer, MobileNet, GhostNet, AlexNet, and LeNet. It also examined optimization methods and explainable AI approaches. The examined research focused on the detection of colorectal cancer by histological and endoscopic images using datasets such as Kather-CRC-2016, CRC-TP, HunCRC, PanNuke, TCGA-COAD, DeepPath, and GEO-CRC. The results showed that deep learning methods have led to significant improvements in the detection of colorectal cancer. However, there are still substantial challenges surrounding data heterogeneity, model diversity, interpretability, clinical applicability, performance harmonization and computing efficiency. Addressing these limitations is critical for building more robust, generalizable, and generally adopted therapeutic applications. Yousaf et al. proposed an enhanced U-Net-based convolutional neural network (CNN) model for brain tumor and ischemic stroke detection [5]. The model was trained on a new dataset created by combining the BRATS 2015 brain tumor dataset and the ISLES 2015 ischemic stroke dataset. An accuracy rate of 99.56% was achieved on the combined dataset. However, the study was tested on small number of datasets and disease categories. Additional validation experiments with varied datasets, imaging conditions, and disease types are required to establish the generalizability of the model and its use in wider clinical settings. Richa and Patro proposed a deep learning (DL)-based model that combines a Convolutional Neural Network (CNN) architecture, feature selection, and feature fusion methods to enhance the accurateness of early breast cancer detection [6]. The model was tested with a dataset of 2800 samples and achieved an accuracy of 94.93%. Due to the large dataset and high computational requirements, its generalizability to different imaging types is limited.

Oral malignancies are a serious global health problem, and early diagnosis is associated with better treatment results and survival, and may allow for greater preservation of oral function and quality of life [7]. Oral cancers are mostly developed from squamous epithelial tissues of oral cavity and can be found in places such as tongue, floor of mouth, buccal mucosa, gingiva, palate and lips [8]. Tobacco and alcohol use, betel quid chewing and HPV infection are established risk factors but poor oral hygiene has also been linked to an increased risk of oral cancer [9]. Clinically, oral cancer may manifest as a non-healing ulcer, red or white lesions, abnormal tissue growth, discomfort, bleeding, trouble in swallowing and changes in speech [10]. Standard diagnostic approaches are clinical examination, imaging assessment (if indicated) and histological examination of biopsy samples. These procedures may be time-consuming and often hampered by sampling problems and inter-observer variability [11].

The resemblance of early-stage lesions to benign formations may lead to delayed diagnosis. Therefore, deep learning-based artificial intelligence approaches have been increasingly used in recent years to support the automatic and reliable classification of oral cancer images. These systems can learn complex patterns from histopathological and clinical images, thereby helping to identify suspicious lesions, differentiate between malignant and benign tissues, and improve the consistency of diagnostic assessments. Oral cancer diagnosis has attracted considerable research attention, with numerous studies investigating the use of machine learning and deep learning architectures to improve its early and accurate detection. Sundari and Maheswari proposed a new deep learning-based diagnostic framework to improve the early detection performance of oral cancer [12]. By considering various imaging conditions, lesion structural complexity, and related factors, the proposed method aims to automatically and accurately categorize benign and malignant lesions. The Contrast-Limited Adaptive Histogram Equalization (CLAHE) technique improves image quality and enables more successful detection of lesions in low-contrast intraoral images. The Xception deep learning architecture serves as the foundation for the suggested model. Nevertheless, in contrast to the traditional Xception architecture, a second convolution layer that was created especially for the middle flow portion of the architecture has been incorporated in order to learn more complex and unique properties. Furthermore, a channel attention mechanism has been incorporated into the architecture to enhance the model’s classification and lesion localization capabilities. Raval and Undavia proposed a deep learning-based detection system for the early diagnosis of skin and oral cancer [13]. The study analyzes different convolutional neural network (CNN) architectures, such as AlexNet, VGGNet, Inception, ResNet, and DenseNet, in detail and compares their performance. To improve model performance, filtering techniques and data augmentation methods were integrated into the system during image preprocessing. Experimental results showed that the DenseNet architecture achieved higher performance than other CNN models. Training accuracy, training loss, and other metrics were used to assess the effectiveness of the suggested approaches. The results showed that skin cancer images achieved high accuracy. At the same time, the oral cancer dataset performed worse due to its limited sample size and the structural complexity of the images. Huang et al. performed oral cancer detection based on an optimized convolutional neural network (CNN) architecture [14]. To improve the performance of the CNN architecture and overcome its structural limitations, a hybrid optimization approach combining the Seagull Optimization Algorithm (SOA) and Particle Swarm Optimization (PSO) was proposed. This optimization process was applied to encompass the structural design, hyperparameters, and training process of the CNN architecture. Furthermore, noise reduction and contrast enhancement steps were integrated into the image preprocessing stage to improve image quality and increase detection performance. The performance of the proposed method was evaluated comparatively with methods such as Fuzzy C-Means (FCM), classical CNN, R-CNN, and ResNet-101. Experimental results show that the proposed approach achieved 96.94% accuracy, 94.65% precision, 91.60% recall, and 88.55% F1-score on the Oral Cancer Image (OCI) dataset. Chang et al. used Raman spectroscopy and many deep learning methods, such as AlexNet, VGGNet, ResNet50, MobileNetV2, and Transformer, to diagnose oral cancer [15]. Empirical evidence demonstrated that the proposed methodology may immediately extract high-level distinguishing features from Raman spectra, obviating the need for manually created features. The study used 16,200 Raman spectra from tongue, gum, and cheek squamous cell carcinomas and their normal counterparts. The ResNet50 model performed the best, with 92.81% accuracy and 92.93% precision, according to comparative analyses of all the models. Welikala et al. generated a large-scale oral cancer dataset by collecting clinical data from various countries to automatically detect early-stage oral potentially malignant disorders, as part of the MeMoSa project [16]. This study presents an oral cancer classification and lesion detection framework based on ResNet-101 and Faster R-CNN, using this dataset. The proposed method achieved an 87.07% F1-score in lesion detection, and the study’s performance was validated with experimental results. Huang et al. proposed an improved Squirrel Search algorithm to find oral cancer automatically and accurately [17]. This work utilized three distinct preprocessing techniques—gamma correction, noise reduction, and data augmentation—to enhance the quality of intraoral images and reduce the issue of inadequate data. A convolutional neural network (CNN) was used for the classification of the processed images, and the Improved SSA method was used to overcome the local minimum issue of classical gradient-based learning. The developed optimization algorithm optimized the layers, neurons, weights, and hyperparameters of the CNN architecture, providing an effective framework. The technique was assessed using a conventional oral cancer dataset and compared with other CNN and artificial intelligence methodologies documented in the literature. The experimental results demonstrated that the proposed technique exhibits superior accuracy and enhanced diagnostic reliability. Das et al. developed a segmentation algorithm, CART, for oral cancer images [18]. The proposed method achieved an accuracy of 83.80%. However, its applicability to a wider range of lesion classifications is limited, as it was developed for a specific lesion classification. A hybrid method called ABC-PSO was developed for oral cancer classification [19]. This method was comparatively examined with the BLDA classifier. It was reported that the developed hybrid method achieved higher accuracy. M. Shamim et al. achieved an 84.72% success rate in oral cancer classification using SVM and infrared (IR) images [20]. Han et al. used the ResNet152 architecture for cancer detection from clinical images [21]. Talwar et al. achieved an 84% F1 score in oral cancer using DenseNet201 and Swin Transformer-based models [22]. Fu et al. investigated the effectiveness of active learning and reinforcement learning in oral cancer detection [23]. Experimental results showed that the active learning performed better than the reinforcement learning. In most of the studies focusing on disease detection from medical images, optimization techniques are widely used to improve the performance of methods employed in preprocessing [24], feature selection [25], machine learning [26], and deep learning stages [27,28]. The use of optimization strategies, particularly in the processes of optimally determining model parameters, performing feature selection, and designing effective network architectures, directly and critically impacts the success of applications. These algorithms enable significant improvements in classification accuracy while simultaneously establishing a balance between computational cost and model complexity.

In addition, artificial intelligence and deep transfer learning-based oral cancer detection and classification techniques using image processing methods have been developed and applied to datasets with different characteristics. Mira et al. investigated the use of a deep learning algorithm to analyze smartphone-acquired intraoral photographs for the early detection of oral cancer [29]. The study proposed a centered image-capture rule, in which the lesion was positioned at the center of the photograph, together with a resampling strategy designed to reduce image variability and address class imbalance. The collected intraoral images were categorized into five groups: normal mucosa, aphthous ulcer, low-risk oral potentially malignant disorders (OPMDs), high-risk OPMDs, and oral cancer. Using the HRNet-W18 model, the study achieved 83.0% sensitivity, 96.6% specificity, 84.3% accuracy, and an F1-score of 83.6% on the reported test set. Tanriver et al. developed a two-stage deep learning-based framework for the automatic detection and classification of oral lesions in photographic images [30]. In the first stage, lesion regions were detected using the YOLOv5l model. In the second stage, the detected lesion regions were classified as benign lesions, oral potentially malignant disorders (OPMDs), or carcinoma using the EfficientNet-b4 model. In separate experiments, different semantic segmentation and classification models were evaluated. For semantic segmentation, a U-Net model with an EfficientNet-b7 backbone achieved the highest Dice score. For lesion classification, EfficientNet-b4 and Inception-v4 obtained the highest F1-scores; however, EfficientNet-b4 was selected for the final pipeline because of its balanced performance, computational efficiency, and lower complexity. The findings indicate that the proposed system has the potential to support oral cancer and OPMD screening as a low-cost, rapid, and non-invasive tool. However, further validation using larger and more diverse datasets is required before clinical implementation. 

In this study, an iHBA-based method is proposed to enhance the accuracy of oral cancer detection. In the proposed framework, input images are first enhanced using Contrast-Limited Adaptive Histogram Equalization (CLAHE) to improve visual quality and resolution. The enhanced images are then resized and forwarded to the ResNet50 architecture for deep feature extraction. Although deep learning-based feature extraction provides highly discriminative representations, the resulting high-dimensional feature space significantly increases computational costs and model complexity. Therefore, an effective feature selection strategy is required to reduce the computational time of the model. Despite being a recently introduced and powerful metaheuristic optimizer, the Honey Badger Algorithm (HBA) may suffer from premature convergence in certain unimodal and multimodal optimization problems due to its susceptibility to local optima and its insufficient balance between exploration and exploitation. To overcome these limitations, an improved variant, namely iHBA, is introduced. In the proposed enhancement, the distance vector formulation used in both the honey mode and digging mode of HBA is reconstructed to strengthen the balance between exploration and exploitation, thereby improving convergence behavior and overall optimization performance. Secondly, a migration strategy based on the Cauchy mutation is incorporated into the proposed framework. The performance of the iHBA algorithm is first evaluated on widely used unimodal and multimodal benchmark functions. The experiments are conducted comparatively against several recent and competitive optimization algorithms, including the Black-Winged Kite Algorithm (BKA), Dandelion Optimization (DO), and the Goose Algorithm (GOOSE). After validating its robustness on benchmark functions with diverse characteristics, the biHBA method is proposed for the feature selection process. In this method, the iHBA method is discretized using a specially designed transfer function to enable the selection of informative features extracted from oral cancer images. The effectiveness of the proposed feature selection approach is assessed through comprehensive experiments, including comparisons with similar metaheuristic algorithms as well as wrapper-based, filter-based, and embedded feature selection methods. A detailed analysis is performed to identify the most suitable feature selection paradigm for the given dataset and problem structure. This stage ensures more efficient utilization of high-dimensional deep features and facilitates the identification of the most discriminative features contributing to the final model performance.

## 2. Materials and Methods

### 2.1. Dataset

**Figure 1:**
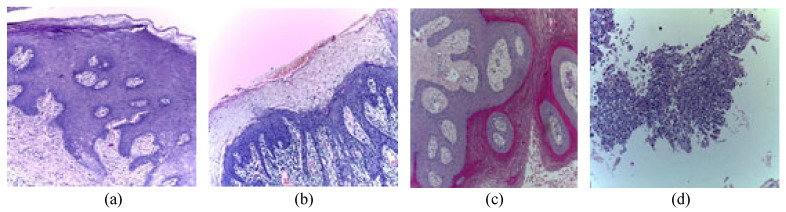
Example images from the Histopathological Imaging Database with 100× magnification level for (**a**,**b**); Normal, and (**c**,**d**); OSCC classes.

**Figure 2:**
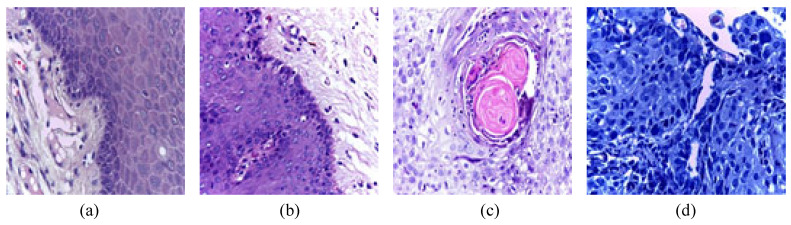
Example images from the Histopathological Imaging Database with 400× magnification level for (**a**,**b**); Normal, and (**c**,**d**); OSCC classes.

The proposed model was evaluated based on Histopathological Oral Cancer Imaging Dataset, which is a publicly available benchmark dataset for oral cancer detection [31]. The Histopathological Imaging Database contains 1224 histopathological oral tissue images of 230 patients. The images are taken from Hematoxylin and Eosin (H&E)- stained biopsy slides, recorded using a Leica DM750 microscope (Leica, Wetzlar, Germany) and an ICC50 HD camera (Leica, Wetzlar, Germany). The dataset is divided into two subsets based on magnification level: 100× and 400×. The 528 images taken at 100× magnification are the first subset. 89 images are of normal oral epithelium, and 439 are of Oral Squamous Cell Carcinoma (OSCC). These images are particularly helpful for tissue-level or architectural analysis, feature extraction, image segmentation, and classification. The second group has 696 images captured under 400× magnification. This group includes 201 images of normal epithelium and 495 images of OSCC. These high-resolution images enable tissue-level and cell-level analysis, particularly for examining nuclear changes and cell texture. A part of this group (269 images) has been used in previous research to classify tissue according to texture features with highly accurate classification. All the images are 2048 × 1536 pixels and in JPEG format. Figure 1 and Figure 2 show the sample images from 100× and 400× magnification levels and OSCC and normal classes.

### 2.2. Proposed Method

**Figure 3:**
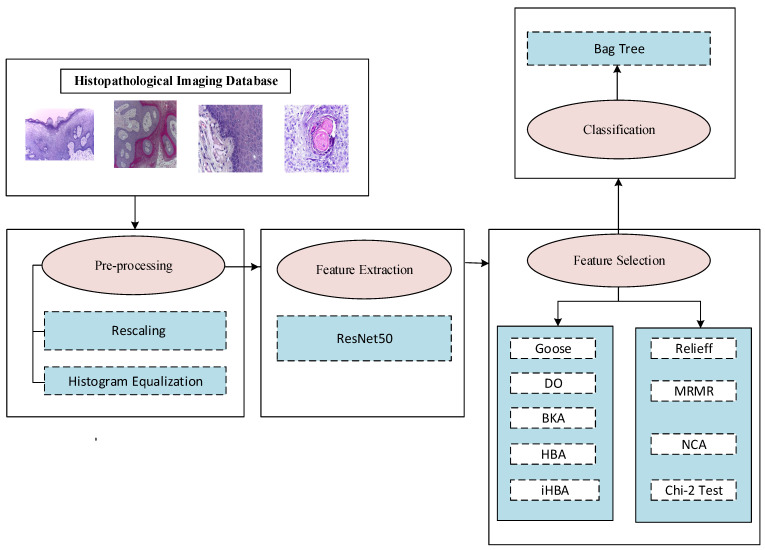
Flowchart of the iHBA-based oral cancer detection.

This study proposes an effective prediction framework for automated oral cancer diagnosis. In the developed method, deep features are extracted using the ResNet50 network architecture, and then the most distinctive features are identified using an improved Honey Badger Algorithm (HBA)-based feature selection process. This significantly improves the model’s classification performance and computational efficiency. The flowchart of the proposed method is presented in Figure 3.

As shown in Figure 3, the images from the Histopathological Imaging Database were resized to the network input size of 224 × 224 × 3. Histogram equalization was applied to enhance the contrast of the resized images. These preprocessing steps made the images suitable for the deep learning network. After the preprocessing stage, deep feature extraction was performed using the ResNet50 architecture, and a 2048-dimensional deep feature vector was obtained for each image. Although ResNet50-based deep feature extraction provides discriminative representations, the resulting feature vectors may contain redundant or irrelevant information due to their high-dimensional structure. To address this issue, a feature selection step was applied before classification, and the extracted features were comprehensively evaluated using different feature selection strategies, including wrapper-based, filter-based, and embedded-based methods. In this process, the proposed iHBA method was used as an effective wrapper-based feature selection strategy. The iHBA method was developed by improving the original Honey Badger Algorithm through the reconstruction of the distance vector and the integration of a Cauchy mutation-based migration strategy to enhance the exploration–exploitation balance. Before being applied to feature selection, the proposed iHBA method was validated through detailed analyses on unimodal and multimodal benchmark functions. After confirming its optimization capability and robustness on these benchmark problems, iHBA was applied to select the most informative features from the ResNet50-based deep feature set. In this context, the proposed iHBA method was compared with metaheuristic algorithms such as GOOSE, DO, BKA, and HBA, as well as traditional feature selection methods such as ReliefF, MRMR, NCA, and the Chi-square test.

#### 2.2.1. Honey Badger Algorithm

The Honey Badger Algorithm is one of the powerful nature-inspired metaheuristic algorithms, proposed by Hashim et al. in 2022 [32]. This method mimics the foraging behavior of the honey badger, an animal renowned for its highly developed sense of smell, enabling it to locate its prey easily. Honey badgers primarily feed on insects, frogs, turtles, lizards, snakes, birds, eggs, and, of course, honey. Although they have a fondness for honey, they are not particularly successful at finding it on their own. To improve their chances, they often collaborate with honey guides—birds that assist them in locating honey. In the algorithm, the pursuit of various foods is categorized into two modes: excavation mode for general foraging and honey mode for searching for honey with the help of the honey guide bird.

Phase 1: Honey mode (exploration)

In this exploration mode, the honey badger expertly tracks down its prey by following the guidance of the honey guide bird. This unique behavior is illustrating the remarkable adaptability and hunting skills of the honey badger in the wild and precisely modeled by Equation (1):

(1) $$x_{new}=x_{prey}+F\times r_{7}\times \alpha \times d_{i}$$

where x_prey_ denotes the position of the prey (best). r_7_ is a random number, and d is the distance vector between the ith individual and the prey. It is calculated as Equation (2):

(2) $$d_{i}=x_{prey}-x_{i}$$

The variable α represents the density factor, which is updated during each iteration using following equation:

(3) $$\alpha =C_{d}\times e^{\left(\frac{-t}{T}\right)}$$

where t and T represent the current and the total iterations. *C_d_* is a constant number taken as 2 (*C_d_* ≥1). This density factor plays a crucial role in balancing exploration and exploitation, facilitating a smooth transition between these two phases. As the number of iterations increases, the value of α decreases. F determines the direction of the search for the next movement, and determines it as Equation (4).

(4) $$F=\left\{\begin{array}{c} 1\;\;\;\;\;\;\;if\;r_{6}\leq 0.5\;\;\;\;\;\;\\ -1\;\;\;\;\;\;\;\;\;\;else\;\;\;\;\;\;\;\;\;\;\; \end{array}\right.$$

where r_6_ is a randomly generated number.

Phase 2: Digging mode (exploitation)

In this mode, the honey badger moves in a cardioid pattern while approaching its prey. Its strong olfactory abilities also help in updating its position. The equation for cardioid motion based on the smell intensity of the prey is provided in Equation (5).

(5) $$x_{new}=x_{prey}+F\times \beta \times I\times x_{prey}+F\times r_{3}\times \alpha \times d_{i}\times |\cos\left(2\pi r_{4}\right)\times (1-\cos\left(2\pi r_{5}\right))|$$

where β is a constant (β > 1) and taken as 6. r_3_ denotes the random number, and I represents the smell intensity of the prey. For the honey badger to move quickly, the value of I must be large.

(6) $$I=r_{2}\times \frac{s}{4\pi d_{i}^{2}}$$

(7) $$S={\left(x_{i}-x_{i+1}\right)}^{2}$$

where S represents the concentration strength, and r_2_ is a random number.

#### 2.2.2. Proposed Honey Badger Algorithm

**Figure 4:**
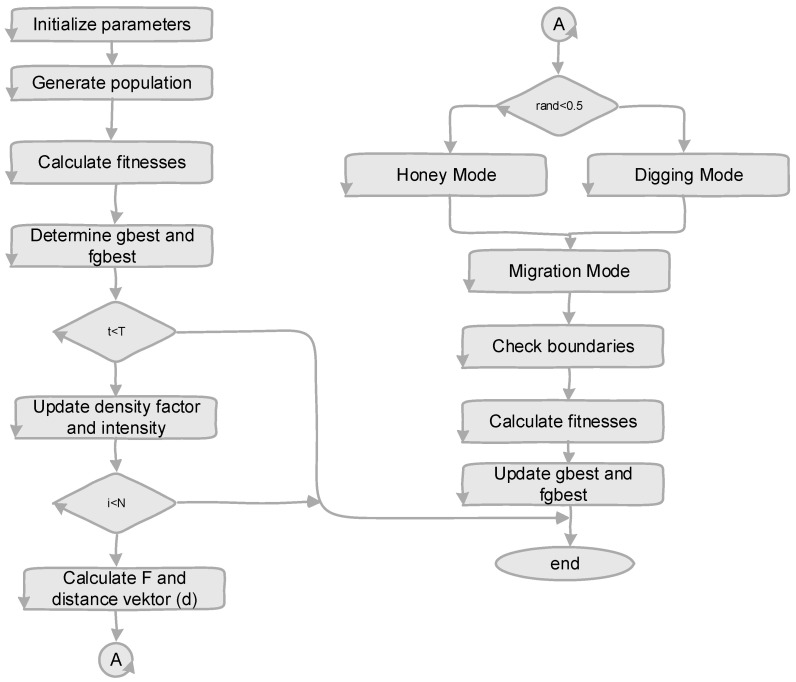
Flowchart of the hybrid honey badger algorithm.

The proposed method is developed to prevent the original HBA optimization from prematurely falling into local optima and to enhance the exploration process. This method addresses some of the shortcomings of the original HBA by integrating the migration strategy of a newly developed black-kited algorithm, which possesses robust search capabilities. Additionally, the distance vector of the HBA is reconstructed for the balancing exploration and exploitation phases. A flow diagram illustrating the developed hybrid Honey Badger Algorithm is presented in Figure 4.

As can be seen in Figure 4, firstly, the parameters of the HBA are initialized. These parameters are included: lower bound, upper bound, dimension of the problem, population size, maximum number of iterations, and β value (determines the ability of HB to get the food). The initial population is generated according to the dimension of the problem and the size of the population. The fitness value of every individual is calculated, and the best individuals are selected according to fitness values. The best individual and its fitness are assigned as the gBest and fgBest.

The algorithm performs the following operations within the loop. At each iteration, the density (α) and intensity (I) factors are calculated as Equations (8) and (9):

(8) $$\alpha =c\times e^{\left(\frac{-t}{T}\right)}$$

(9) $$I=r_{2}\times \frac{s}{4\pi d_{i}^{2}}$$

Then, by selecting either the honey or digging mode, the new population positions are updated via Equations (10) and (11).

(10) $$x_{new}=x_{prey}+F\times r_{7}\times \alpha \times d_{i}$$

(11) $$x_{new}=x_{prey}+F\times \beta \times I\times x_{prey}+F\times r_{3}\times \alpha \times d_{i}\times |\cos\left(2\pi r_{4}\right)\times (1-\cos\left(2\pi r_{5}\right))|$$

The fitness values of the updated solutions are computed and compared with their previous values, and the better solutions are retained. To improve the exploration capability of the method, the solutions are moved into a migration mode to enable searching in different locations. In this mode, the Cauchy distribution operator is first calculated using Equation (12).

(12) $$c=tan[\left(\delta -0.5\right)\pi ]$$

Then, based on this parameter and the prey’s location, new solutions are generated as Equations (13) and (14):

(13) $$x\left(t+1\right)=x\left(t\right)+c\times \left(x\left(t\right)-x_{prey}\right)$$

(14) $$x\left(t+1\right)=x\left(t\right)+c\times (x_{prey}-mx\left(t\right))$$

The newly generated solutions are compared with the previous ones, and the best solutions are selected. The solution boundaries are checked, and if a solution lies outside the search space of the problem, it is pulled back into the feasible range. This loop continues until the stopping criterion is satisfied, and the gbest value is returned as the best solution for the problem.

As a result of the investigations, it was observed that the HBA method did not exhibit sufficient performance in some unimodal and multimodal test functions. Accordingly, in order to improve both exploration and exploitation capabilities and to make the exploration–exploitation balance of the algorithm more effective, the distance vector used in the HBA method was redefined. The distance vector used in honey mode and digging mode is redefined as Equation (15).

(15) $$d_{i}=|x_{prey}-x_{i}|$$

The absolute value of the distance between the honey badgers was calculated, focusing solely on the magnitude of this distance. Next, the approach to and departure from the prey were determined using the specified flag value, labeled as F. F is calculated as Equation (16).

(16) $$F=\left\{\begin{array}{c} 1\;\;\;\;\;\;\;if\;r_{6}\leq 0.5\;\;\;\;\;\;\;\\ -1\;\;\;\;\;\;\;\;\;\;else\;\;\;\;\;\;\;\;\;\;\; \end{array}\right.$$

The parameter F determines the direction of the search, and the next movement is updated as Equation (17).

(17) $$x_{new}=x_{prey}+F\times r_{7}\times \alpha \times d_{i}$$

where r_7_ is a random number. Additionally, a migration strategy is incorporated into the algorithm to improve convergence behavior and enhance the transition balance between the exploration and exploitation phases. The migration strategy is based on the Cauchy mutation, a random mutation process derived from the Cauchy distribution. This approach has been demonstrated in multiple studies to be effective in avoiding local optima [33,34,35,36]. The cumulative distribution function of the Cauchy distribution is provided as Equation (18).

(18) $$F\left(x\right)=\frac{1}{2}+\frac{1}{\pi }arctan(x)$$

Cauchy mutation enhances the search process by enabling individuals to undergo significant mutations, which allows exploration in more distant areas of the solution space. This approach improves the method’s global search capability and helps prevent it from becoming trapped in local optima. The equations of motion for the position of the honey badger are formulated as Equations (19) and (20):

(19) $$x\left(t+1\right)=x\left(t\right)+c\times \left(x\left(t\right)-x_{prey}\right)$$

(20) $$x\left(t+1\right)=x\left(t\right)+c\times (x_{prey}-mx\left(t\right))$$

where $x\left(t\right)$ and $x\left(t+1\right)$ are the current and the next positions of the badgers, respectively. $x_{prey}$ represents the best position found so far. m and c parameters are calculated via Equations (21) and (22):

(21) $$m=2\sin\left(r+\frac{\pi }{2}\right)$$

(22) $$c=tan[\left(\delta -0.5\right)\pi ]$$

c represents the number generated from the Cauchy distribution. r and δ are taken in the range of (0,1). The behavior of the m parameter determines the exploration and exploitation phases.

#### 2.2.3. Binary iHBA Based on a Sin-Cos Transfer Function

**Figure 5:**
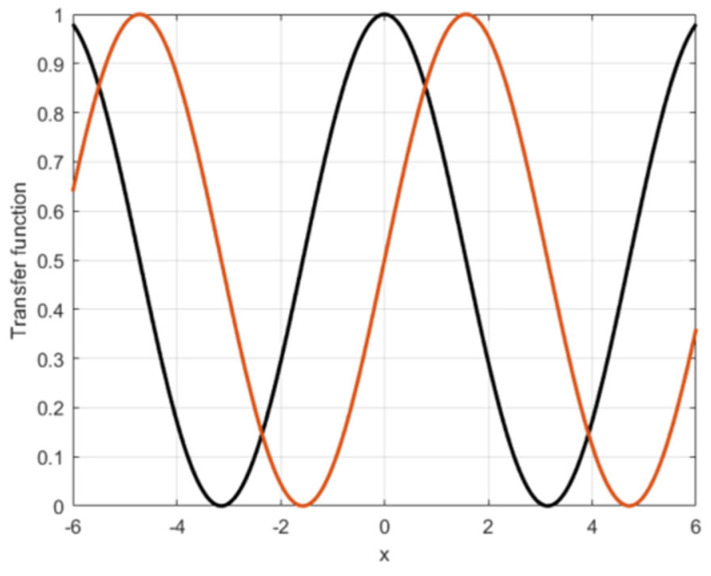
Sine and cosine functions.

This section presents a combined novel transfer function designed to convert the continuous-time position values of honey badgers into binary values. Utilizing sine and cosine transfer functions to convert the HBA method into a binary format increases the diversity of the generated population. The combined sine and cosine transfer function [37] proposed by Sharifian et al. requires significant time to compute and select fitness values at each iteration. Given the importance of low time complexity in feature selection, this study introduces a new method for discretization and feature selection that utilizes sine and cosine transfer functions. The changes of the sine and cosine functions are presented in Figure 5.

According to the cosine transfer function, the conversion from the continuous search space to the binary search space is performed using Equations (23) and (24):

(23) $$B_{1}=\frac{cos(x_{i,j})}{2}+0.5$$

(24) $$x_{i,j}^{1}=\left\{\begin{array}{c} 1,\;\;\;\;if\;r_{1}<B_{1}\\ 0,\;\;\;\;if\;r_{1}\geq B_{1} \end{array}\right.$$

where B1 represents the new binary position created by Cos(.) transfer function. xi,j is the current position of the ith honey badger in the jth dimension. r1 denotes the random numbers in the range of (0,1). $x_{i,j}^{1}$ represents the binary value created with cosine transfer functions. Similarly, continuous position values are transformed into binary form using the sine transfer function, as Equations (25) and (26):

(25) $$B_{2}=\frac{sin(x_{i,j})}{2}+0.5$$

(26) $$x_{i,j}^{2}=\left\{\begin{array}{c} 1,\;\;\;\;if\;r_{2}<B_{2}\\ 0,\;\;\;\;if\;r_{2}\geq B_{2} \end{array}\right.$$

where B_2_ represents the new binary position created by Sin(.) transfer function. r_2_ denotes the random numbers in the range of (0,1). $x_{i,j}^{2}$ represents the binary value created with cosine transfer functions. Afterwards, features that are not selected by both transfer functions are assigned a value of zero (Equation (27)).

(27) $$x_{i}=\left\{\begin{array}{c} 0\;\;\;\;\;if\;x_{i,j}^{1}=0\;||\;x_{i,j}^{2}=0\;\;\;\;\;\;\;\;\;\;\\ 1,\;\;\;\;\;otherwise\;\;\;\;\;\;\;\;\;\;\;\;\;\;\;\;\;\;\;\;\; \end{array}\right.$$

In this way, irrelevant features that are jointly unselected by both transfer functions are eliminated.

## 3. Experiments and Results

Comprehensive experimental studies were conducted on standard benchmark functions with varying difficulty levels and mathematical properties to show the performance of the proposed method from various perspectives. A total of 10 benchmark functions, five unimodal and five multimodal, were selected, all of which are widely used in the literature. Using both separable and non-separable types of functions, the method’s exploitation and exploration capabilities were evaluated in various scenarios. HBA was comparatively examined with optimization methods developed around the same time, such as BKA, DO, GOOSE, and the original HBA. The HBA-specific parameter settings used in the experimental studies, as well as the parameters common to all algorithms, are detailed in Table 1. Values recommended in the literature were taken into account in determining the parameter values, ensuring a fair comparison across all methods [32].

Detailed information about the used benchmark functions is presented in Table 2 and Table 3, respectively. The tables also include the mathematical formulation of each test function, the parameter ranges determined for the search space, and the best (optimal) solution values known within these ranges. This allows for an objective and comparable analysis of the method’s performance in both single-peak (unimodal) and multimodal complex search environments.

As shown in Table 2 and Table 3, benchmark functions with different characteristics were used to analyze the behavior of the algorithms in a comprehensive and multifaceted manner. To this end, a set of 5 unimodal and 5 multimodal test functions, frequently referenced in the literature, was selected. When selecting the functions, not only the diversity between unimodal functions representing simple search spaces with a single vertex and multimodal functions with multiple local minima, but also their separable and inseparable structures were considered. This enabled an objective, comparable, and detailed evaluation of the algorithms’ performance in both low- and high-complexity search environments, with and without variable interaction. This comprehensive set of functions enables a clearer demonstration of the strengths and weaknesses of the proposed method across different problem types. The proposed method was then applied to improve the performance in classifying images in the Histopathological Imaging Database created for oral cancer detection.

The hyperparameters of the proposed method were determined by considering the architectural requirements of the models used, commonly adopted settings in the literature, and the parameter values recommended in the original algorithms. In the preprocessing stage, CLAHE was applied to the green channel to enhance local contrast. The NumTiles parameter was set to [8 8], dividing each image into 64 local regions, and the NBins parameter was set to 128 for histogram representation. In this study, the input images to the pre-trained ResNet50 model were rescaled to the standard input size of 224 × 224 × 3, as required by the ResNet50 architecture. Features were extracted from the average pooling layer, yielding 2048 features per image. In the classification phase, the Bagged Trees method was created using decision trees as the basic learner. The number of learning cycles was determined as 100 to obtain a sufficiently stable ensemble structure while keeping computational cost at a reasonable level [38,39].

The hyperparameter values of the proposed optimization method were selected by considering the values recommended in the original Honey Badger Algorithm study [32]. The C parameter, one of the parameters of the HBA method, represents the constant that controls the density factor, and in this study, C = 2 was taken. In the Honey Badger Algorithm, this parameter is used to regulate the balance between exploration and exploitation throughout the search process. The density factor decreases as the iterations progress, allowing the algorithm to perform a broad search initially and, in later stages, to perform a more intensive search in regions closer to the best solution. The beta parameter represents the honey badger’s ability to reach food, and in this study, β = 6. In HBA, the beta value influences the behavior of candidate solutions as they move towards the prey/food location. This parameter helps the algorithm to perform a more efficient search in the solution space and to control its ability to orient itself towards the best solution.

The proposed iHBA method was examined in a comparative analysis with the Black-Winged Kite Algorithm (BKA) [40], Dandelion Optimization (DO) [41], Goose Algorithm (GOOSE) [42], and the original HBA [32] algorithms, all of which were developed in the literature during a similar period and are widely used among current heuristic search approaches. The primary objectives of the comparison are to evaluate the competitive performance of the iHBA in comparison to other contemporary optimization methods and to demonstrate its tangible advantages over classical HBA. It enables a thorough examination of the algorithm’s behavior across various function types and the degree to which it enhances the exploration–exploitation balance.

### 3.1. Results on Experimental Set-1: Benchmark Functions

To ensure a fair and reliable comparison in the experimental evaluations, each algorithm was tested with 20 independent runs on each benchmark function. The multiple-run strategy helped control for variation arising from the algorithms’ random components and ensured greater statistical significance of the results. Performance distributions were detailed by calculating the minimum, maximum, average, and standard deviation of the outputs obtained from each run.

The results obtained from the test functions (Table 4 and Table 5) show that the HBA method performs better on multimodal functions than on unimodal ones. This demonstrates that the algorithm’s exploration ability is relatively strong, but its exploitation ability is relatively weak. For certain function types, this imbalance between exploration and exploitation restricts the HBA method’s capacity to find optimal solutions. By enhancing the exploration–exploitation balance of HBA method’s, the iHBA approach was put forth to solve this flaw. Through enhancements such as the redesign of the distance vector and the Cauchy mutation-based migration strategy, iHBA not only increases search diversity but also utilizes existing good solutions more efficiently.

Comparative analyses show that iHBA produces better results on unimodal functions than all competing algorithms. On multimodal functions, iHBA is observed to perform competitively with the BKA and DO algorithms. The outcomes reveal that the suggested method can provide consistent and reliable performance in both single-peak and multi-peak problem environments, while also improving the exploration–exploitation balance more effectively.

### 3.2. Results on Experimental Set-2: Histopathological Imaging Database for Oral Cancer

Images from the Histopathological Imaging Database for oral cancer detection dataset were preprocessed before being fed into the deep learning model. In this stage, we first analyzed the images for contrast imbalances and regional light-darkness differences that could negatively impact recognition performance. To address these issues and enhance the clarity of textural details, the Contrast-Limited Adaptive Histogram Equalization (CLAHE) method was applied. In the second stage of preprocessing, all histopathologic images were resized to match the input dimensions of the deep learning model. Experimental studies conducted on this dataset were designed in two separate stages to demonstrate the superiority of the iHBA method in the feature selection process.

#### 3.2.1. Experimental Results Without Feature Selection Processes

The first stage is a basic reference scenario that does not use any feature selection. This stage aims to demonstrate the classification performance of the features used without any selection and to establish a comparable basis for improvements in the second stage. To this end, 2048 deep features were extracted from all images in the dataset using the ResNet50 deep network architecture. The goal of the robust representational capabilities and extensive layer structure of the ResNet50 was to extract both high-level and low-level structural information from the images. The training and assessment phases were separated by randomly dividing the dataset into 80% for training and 20% for testing after feature extraction. The description and division of the Histopathological Imaging Database are presented in Table 6 for 100× and 400× magnification factors.

**Figure 6:**
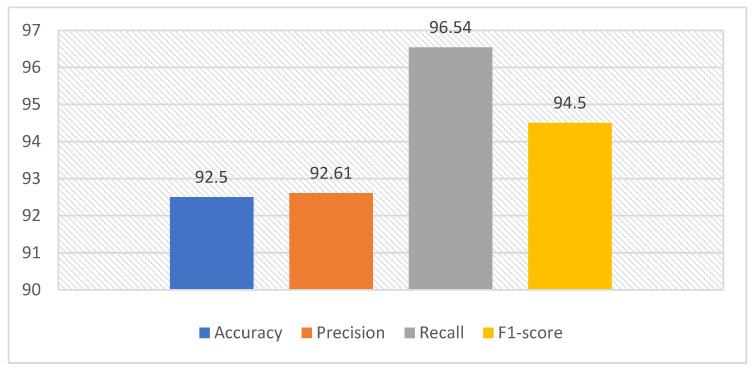
Performance evaluation metrics for a 100× magnification factor.

**Figure 7:**
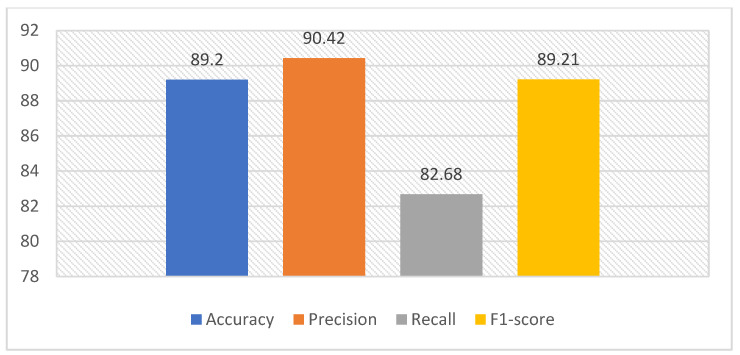
Performance evaluation metrics for a 400× magnification factor.

In the classification stage, a Bagged Tree-based ensemble classifier was chosen to distinguish between Normal and OSCC classes reliably. This method is based on the principle of using multiple decision trees created from the same dataset with different sampling strategies. After the training process was completed, the model was run on the test data to obtain predictions. The predicted outcomes were compared with the real labels to measure performance. For this evaluation, a confusion matrix was first created. Comprehensive performance metrics based on accuracy, precision, recall, and F1-score were then calculated based on these values. This first-stage evaluation serves as the baseline performance without feature selection. It provides the basis for an objective comparison of the impact of the improvements to be implemented in the second stage. In this context, the model was run five times without the feature selection stage, and the results from each run were recorded. The outputs from the results for 100× and 400× magnification factors of the oral cancer histopathological images are presented in Figure 6 and Figure 7, respectively.

**Figure 8:**
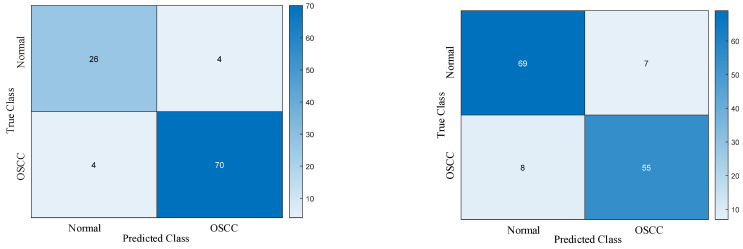
Confusion matrices for Normal and OSCC classes without feature selection for 100× and 400× magnification factors, respectively.

The confusion matrices generated for the best-performing Normal and OSCC classes, obtained from five independent runs, are presented in Figure 8 for 100× and 400× magnification factors, respectively.

#### 3.2.2. Experimental Results with Feature Selection Processes

Although features from the ResNet50 architecture produced good classification performance, as the figure illustrates, a major disadvantage is the comparatively large size of the extracted feature set. Higher computing costs, a longer learning curve, and a greater chance of overfitting can result from a high-dimensional feature space. To improve this situation and obtain a more compact and meaningful feature set, a feature selection stage was added to the model. In this additional stage, a comprehensive evaluation was conducted using both metaheuristic-based feature selection algorithms and traditional statistical/filter-based methods to determine which method yields the more effective, optimized feature subset.

Experimental results for 100× magnification factor: The proposed iHBA method has been extensively compared with its counterparts, the BKA, DO, Goose, and original HBA optimization methods. In this context, each algorithm was tested with five runs, and the mean, minimum, maximum, and standard deviation (std) were calculated. Thus, both the general success trends and the stability levels of the methods could be evaluated. All statistical results are presented in detail in Table 7, clearly showing the performance differences between the methods.

**Figure 9:**
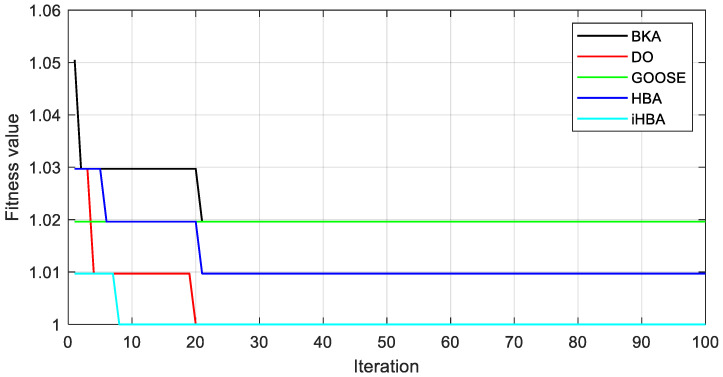
Convergence curves of recent metaheuristics for a 100× magnification factor.

When the results presented in Table 7 are examined, it is clearly seen that the proposed method exhibits superior performance in terms of mean, maximum, minimum, and standard deviation (std/mean) compared to the BKA, Goose algorithm, and original HBA. These results show that the proposed method is more effective than competing methods in terms of both average solution quality and solution stability. In addition, the suggested method performs competitively with the DO method. In evaluating optimization algorithms, not only the final solution quality but also the behavior of the algorithm during the search process is of great importance. In this context, convergence curves are extremely important in showing how quickly algorithms approach the optimum solution, to what extent they can balance exploration and exploitation, and the level of stability in the search process. Therefore, the convergence curves obtained to examine the convergence behavior of the proposed method and other comparative methods in the feature selection phase of the prediction model proposed for the oral cancer dataset are presented in Figure 9. These curves allow for a visual comparison of the speed at which the algorithms reach a solution, enabling a clearer assessment of the proposed method’s performance superiority.

Compared with other algorithms, the suggested iHBA approach shows faster, more stable convergence, as shown in the figure. iHBA exhibits stable behavior by rapidly reaching its lowest level and maintaining it throughout the iteration by gradually decreasing the fitness value from the initial iterations. The DO algorithm also shows relatively fast convergence, reaching a low fitness value after a certain number of iterations; however, this value remains above the result obtained by iHBA. HBA, while providing gradual improvement, converges more slowly than iHBA and stabilizes at a higher minimum value. On the other hand, the Goose algorithm progresses with an almost constant fitness value throughout the iteration and does not show significant improvement. This indicates that the method is stuck at a local minimum in the problem space or does not offer sufficient exploration capacity. The weakest performance belongs to the BKA method, as it progresses with a high initial fitness value and reaches early saturation in a low-quality solution.

Experimental results for 400× magnification factor: The proposed method was also extensively evaluated on a dataset created by applying 400× magnification to oral cancer images. Table 8 presents the statistical performance metrics obtained from five independent runs of other optimization methods, compared with those of the proposed approach.

**Figure 10:**
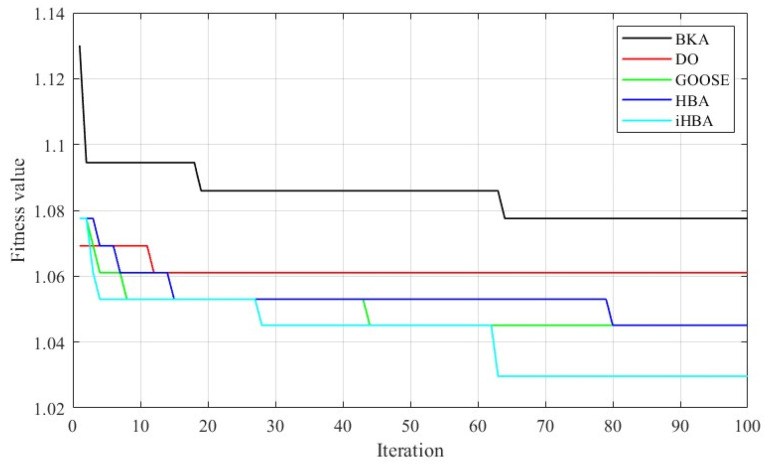
Convergence curves of recent metaheuristics for a 400× magnification factor.

Examining the results in Table 8, it is clearly seen that the proposed method exhibits superior performance compared to all comparative methods. This finding confirms that the method has a strong optimization capability in terms of both solution quality and stable convergence behavior. Convergence curves of recent metaheuristics for a 400× magnification factor of the oral cancer dataset are presented in Figure 10.

The convergence curves presented in Figure 10 clearly show that the proposed iHBA method exhibits faster and more stable convergence performance compared to other optimization algorithms. It is observed that iHBA gradually and steadily decreases its fitness value from the initial iterations, reaching its lowest fitness value after approximately the middle iterations, and maintaining this superiority for the rest of the process. Among the compared algorithms, the BKA method shows the highest fitness values, but its convergence speed is quite slow. Although the DO algorithm and GOOSE exhibit quicker initial convergence, they fall short of iHBA’s minimum fitness level. The limited improvement capability of the traditional HBA approach is evident in its tendency to get stuck at a higher fitness level than iHBA. Overall, the curves reveal that iHBA has a superior optimization ability compared to other methods in terms of both fast convergence and low final fitness value. This indicates that the method develops a more effective search strategy and more successfully navigates towards the optimum in the solution space. Table 9 presents a performance comparison of meta-heuristic-based feature selection algorithms in terms of accuracy, recall, precision, and F1-score for a 100× magnification factor of the dataset.

Examining the results presented in Table 9, the proposed iHBA method achieved higher values in all metrics of accuracy, recall, precision, and F1-score compared to other meta-heuristic algorithms (MAs) included in the comparison. For further evaluation for the proposed method, various feature selection methods, namely wrapper-based, filter-based, and embedded-based methods, are performed for the classification of the oral cancer. The results of the feature selection are given comparatively in Table 10 with Relieff, Minimum Redundancy Maximum Error (MRMR), Neighborhood Component Analysis (NCA), and Chi-Square (χ^2^) Test. Methods are evaluated in terms of accuracy, precision, recall, specificity, F1-score, and number of selected features.

**Figure 11:**
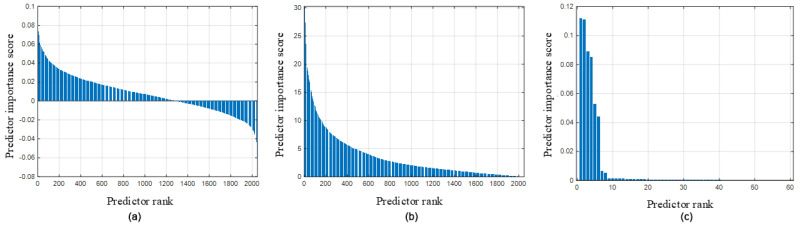
Predictor importance scores for (**a**) Relieff, (**b**) Chi2, (**c**) MRMR feature selection methods for a 100× magnification factor.

**Figure 12:**
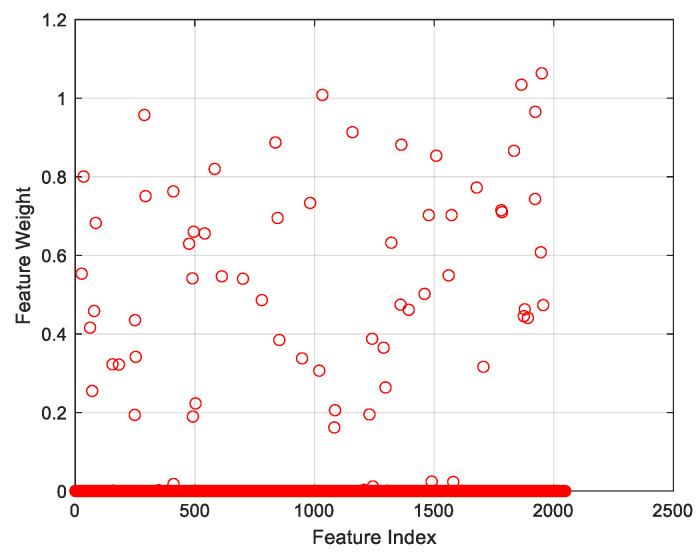
Feature weight for the NCA feature selection method for a 100× magnification factor.

As shown in Table 10, the iHBA-based feature selection approach achieves a very high classification performance when integrated into the oral cancer diagnostic system. Compared to other methods, it clearly demonstrates significant advantages in accuracy, precision, recall, and F1-score. Furthermore, when examining the number of selected features, it is noteworthy that the MRMR and NCA methods achieve acceptable levels of success with a smaller feature set. This result shows that iHBA is a powerful meta-heuristic method for achieving high accuracy. Additionally, MRMR and NCA offer efficient alternatives with smaller feature spaces. The predictor importance scores for (a) Relieff, (b) Chi2, and (c) MRMR feature selection methods for a 100× magnification factor are presented in Figure 11. Also, feature weights graph for the NCA feature selection method is given in Figure 12.

**Figure 13:**
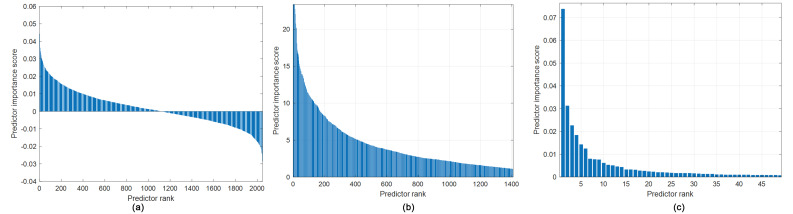
Predictor importance scores for (**a**) Relieff, (**b**) Chi2, (**c**) MRMR feature selection methods for a 400× magnification factor.

**Figure 14:**
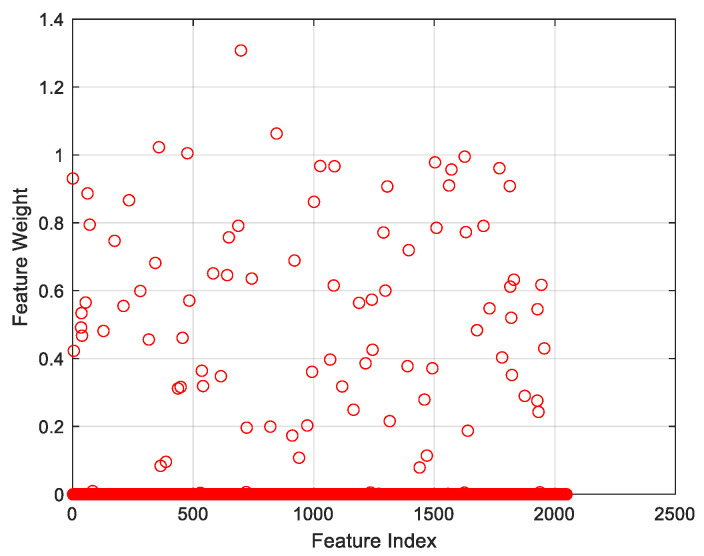
Feature weight for the NCA feature selection method for a 400× magnification factor.

Considering the weighting scores of the ReliefF, Chi-Square (Chi2), and MRMR feature selection methods presented in Figure 11, the number of features to be selected for each method was determined separately. In this context, as a result of examining the weight distributions, 1200 features were selected for the ReliefF method, 100 features for the Chi2 method, and 20 features for the MRMR method. Determining a fixed and universal amount of features for all approaches was deemed meaningless because each feature selection method has different weighting characteristics and evaluation criteria. As a result, the weight vectors of each approach were visually assessed, and the optimal amount of features specific to the applicable method was determined by considering the observed breakpoints and diminishing trends in the weight values. For the NCA method, whose feature weights are presented in Figure 12, features with weight values greater than 0.02 were selected and included in the evaluation. Similarly, feature selection was applied to images magnified 400× in the same version using Relieff, Chi2, and MRMR methods, and the predictor score graphs for these methods are given in Figure 13. Similarly, feature weight for the NCA is presented in Figure 14 for a 400× magnification factor.

Following the feature selection process shown in Figure 13 and Figure 14, the classification of oral cancer images was completed using the steps outlined in Figure 1. In this context, the selected features were given as input to the relevant classification model, and the system’s discrimination performance was evaluated. The classification results are presented in Table 11 and Table 12, and compared with those obtained using other feature selection techniques. Using these tables, the effects of different feature selection approaches on classification performance are analyzed in detail, using key performance metrics such as accuracy, precision, recall, and F1-score. This comparative evaluation clearly demonstrates the effectiveness and superiority of the proposed approach.

ReliefF, MRMR, NCA, Chi-Square (Chi2) Test, HBA, BKA, Goose, and DO methods were used in comparison analyses, and the findings showed that the suggested method excelled all of these approaches in classifying oral cancer photos. The results show a notable improvement in important performance parameters, including F1-score, precision, sensitivity (recall), and classification accuracy.

## 4. Discussion

In this study, the distance vector used in the HBA method was restructured, and a Cauchy-mutation-based migration strategy was integrated to improve the exploration and exploitation capabilities of HBA and achieve a better exploration–exploitation balance. The proposed iHBA method was first evaluated using commonly employed unimodal and multimodal benchmark functions. The obtained results were then comparatively analyzed against those of other powerful optimization algorithms.

Comparative analyses showed that iHBA produced superior results on unimodal functions compared with all competing algorithms. For multimodal functions, iHBA demonstrated competitive performance relative to the BKA and DO algorithms. These findings indicate that the proposed method can provide consistent and reliable performance in both unimodal and multimodal optimization problems while achieving a more effective exploration–exploitation balance. Furthermore, the performance comparisons showed that iHBA outperformed current powerful algorithms in terms of convergence speed, success in reaching the global optimum, and stability. In the second stage, the proposed method was applied to the classification of histopathological images of oral cancer. In this context, an efficient and innovative deep feature-extraction framework was designed, and a robust feature-selection mechanism based on the proposed iHBA method was developed. During the feature selection stage, the performance of the proposed iHBA method was evaluated not only against metaheuristic algorithms in the same class but also against commonly used wrapper-based, filter-based, and embedded-based methods. Through these comprehensive experimental analyses, the most suitable feature selection approach for the dataset and problem structure was identified in detail. The implemented feature selection process enabled a more efficient representation of the high-dimensional and complex deep feature space, reduced the model’s tendency toward overfitting, and improved its generalization ability. Furthermore, it facilitated the identification of the most discriminative features contributing to classification performance.

The experimental results on the oral cancer dataset showed an accuracy of 98.08%, a precision of 100%, a recall of 97.18%, and an F1 score of 98.57% at a magnification factor of 100×. For the 400× magnification factor, an accuracy of 98.08%, a precision of 98.55%, a recall of 98.55%, and an F1 score of 98.55% were achieved. The proposed method was also compared with both CNN-based and transformer-based approaches. For this purpose, features were first extracted using ResNet18 and Vision Transformer (ViT)-based deep learning architectures, and their classification performances were evaluated. Subsequently, the proposed method was compared with the Residual and Transformer-Based Architecture, ResTransNet [43]. The results of the 5-fold cross-validation comparisons with other deep learning architectures on the oral cancer dataset are presented in Table 13.

As shown in Table 13, the results indicate that the suggested method outperforms both the CNN-based method (ResNet18) and the transformer-based method (ViT) in terms of classification performance. Although ResTransNet has a hybrid structure to capture both local and global data, the proposed approach seems to be more effective in identifying discriminative features. This superior performance can be attributed to the proposed method’s ability to select informative features more effectively and to distinguish between classes more accurately.

The suggested technique contributes to the existing literature methods by integrating the deep features with an efficient feature selection process based on iHBA. This structure decreases the dimension of the feature space, while retaining discriminative information and boosting classification performance.

However, although these results are intriguing, there are several limitations to this study. The proposed method was evaluated using a specific histopathological oral cancer image dataset and two magnification factors. Future research may concentrate on testing the suggested framework on different histopathological datasets, integrating explainable artificial intelligence techniques, and evaluating the model in real clinical decision-support scenarios. From a clinical perspective, the proposed approach has the potential to support pathologists as an automated, consistent decision-support tool for the analysis of histopathological images of oral cancer. 

## 5. Conclusions

In conclusion, this study proposed an improved Honey Badger Algorithm, called iHBA, by restructuring the distance vector and integrating a Cauchy-mutation-based migration strategy to enhance the exploration–exploitation balance of the original HBA method. The proposed iHBA was first evaluated on unimodal and multimodal benchmark functions, where it demonstrated superior or competitive performance compared with several powerful optimization algorithms in terms of stability and success in reaching the global optimum.

The proposed iHBA was then integrated into a deep learning-based framework for the classification of histopathological images of oral cancer as a feature selection mechanism. The fusion of ResNet18- and ViT-based deep features enabled the extraction of complementary and discriminative representations, while iHBA-based feature selection reduced the complexity of the high-dimensional feature space and improved classification performance. The experimental results obtained at both 100× and 400× magnification factors indicate that the developed approach provides an effective and competitive solution for the automated analysis of histopathological images of oral cancer compared with existing methods in the literature.

In conclusion, the suggested framework is a potential decision-support strategy that could aid pathologists in analyzing histopathological oral cancer images and enable a more accurate and efficient diagnostic workflow.

## Figures and Tables

**Table 1:** Parameter setting.

Parameter	Definition	Value
Max_iter	Maximum number of iterations	1000
N	Number of the search agent	20
Run	Run_time	20
C	Constant for the density factor	2
Beta	The ability of HB to get the food	6
dim	Dimension	10

**Table 2:** Unimodal benchmark functions.

No.	Function Name	Formula	F_opt_	Type	Range
F_1_	Quartic	$f\left(x\right)=\sum_{i=1}^{D}ix_{i}^{4}$	0	S	[−1.28,1.28]
F_2_	Zakharov	$f\left(x\right)=\sum_{i=1}^{n}x_{i}^{2}+{\left(\sum_{i=1}^{n}0.5\;i\;x_{i}\right)}^{2}+{\left(\sum_{i=1}^{n}0.5\;i\;x_{i}\right)}^{4}$	0	NS	[−5,10]
F_3_	Powell	$f\left(x\right)=\sum_{i=1}^{\frac{n}{k}}\left[{\left(x_{4i-3}+10x_{4i-2}\right)}^{2}+5{\left(x_{4i-1}-10x_{4}\right)}^{2}+{5\left(x_{4i-2}+10x_{4i-1}\right)}^{4}+10{\left(x_{4i-1}-{10x}_{4}\right)}^{4}\right]$	0	NS	[−4,5]
F_4_	Schwefel 2.22	$f\left(x\right)=\sum_{i=1}^{n}\left|x_{i}\right|+\prod_{i=1}^{n}\left|x_{i}\right|$	0	NS	[−10,10]
F_5_	Rosenbrock	$f\left(x\right)=\sum_{i=1}^{D-1}\left[100{\left(x_{i+1}-x_{i}^{2}\right)}^{2}+{\left(x_{i}-1\right)}^{2}\right]$	0	NS	[−30,30]

S and NS denote the separable and non-separable type of the benchmarks.

**Table 3:** Multimodal benchmark functions.

No.	Function Name	Formula	F_opt_	Type	Range
F_6_	Foxholes	$f\left(x\right)={\left[\frac{1}{500}+\sum_{j=1}^{25}\frac{1}{j+\sum_{j=1}^{2}{\left(x_{i}-a_{ij}\right)}^{6}}\right]}^{-1}$	0.9980	S	[−65.536,65.536]
F_7_	Branin	$f\left(x\right)=x_{2}-\left(\frac{5.1}{4\pi^{2}}\right)x_{1}^{2}+\left(\frac{5.1}{\pi }\right){\left(x_{1}-6\right)}^{2}+10\left(1-\frac{1}{8\pi }\right)cos\left(x_{1}\right)+10$	0.3979	S	[−5,10] × [0,15]
F_8_	Bohachevsky1	$f\left(x\right)=x_{1}^{2}+2x_{2}^{2}-0.3cos\left(3\pi x_{1}\right)-0.4cos\left(4\pi x_{2}\right)+0.7$	0	S	[−100,100]
F_9_	Griewank	$f\left(x\right)=\frac{1}{4000}\sum_{i=1}^{n}x_{i}^{2}-\prod_{i=1}^{D}cos\left(\frac{x_{i}}{\sqrt{i}}\right)+1$	0	NS	[−600,600]
F_10_	Ackley	$f\left(x\right)=-20exp\left(-0.2\sqrt{\frac{1}{D}\sum_{i=1}^{D}x_{i}^{2}}\right)-exp\left(\frac{1}{D}\sum_{i=1}^{n}cos\left(2\pi x_{i}\right)\right)+20+e$	0	NS	[−35,35]

S and NS denote the separable and non-separable type of the benchmarks.

**Table 4:** Performance analysis of the proposed in unimodal benchmark functions.

Fun.	Index	İHBA	BKA	HBA	DO	GOOSE
F_1_	Avg	9.2341 × 10^−5^	1.5888 × 10^−4^	1.2508 × 10^−4^	0.0012	0.0069
	Min	6.3787 × 10^−6^	3.7256 × 10^−6^	7.5297 × 10^−6^	2.9549 × 10^−4^	9.0410 × 10^−4^
	Max	2.6891 × 10^−4^	6.5644 × 10^−4^	3.6539 × 10^−4^	0.0035	0.0193
	std	7.9560 × 10^−5^	1.5655 × 10^−4^	1.0172 × 10^−4^	9.8266 × 10^−4^	0.0050
F_2_	Avg	0	5.4688 × 10^−149^	7.7498 × 10^−274^	2.3383 × 10^−12^	6.1871 × 10^−4^
	Min	0	4.9494 × 10^−212^	4.3186 × 10^−284^	1.7034 × 10^−13^	2.2014 × 10^−4^
	Max	0	1.0938 × 10^−147^	1.4926 × 10^−272^	1.6847 × 10^−11^	0.0011
	std	0	2.4457 × 10^−148^	0	3.7173 × 10^−12^	3.0874 × 10^−4^
F_3_	Avg	0	5.4037 × 10^−152^	1.0385 × 10^−127^	1.0819 × 10^−4^	3.2672
	Min	0	9.5657 × 10^−209^	1.3929 × 10^−223^	1.3590 × 10^−5^	0.2507
	Max	0	9.3854 × 10^−151^	2.0770 × 10^−126^	2.4472 × 10^−4^	6.5136
	std	0	2.1060 × 10^−151^	4.6443 × 10^−127^	6.8848 × 10^−5^	1.7902
F_4_	Avg	2.9462 × 10^−281^	2.2977 × 10^−86^	4.1654 × 10^−175^	3.2750 × 10^−8^	3.2672
	Min	1.5784 × 10^−299^	1.5892 × 10^−107^	3.2918 × 10^−181^	8.4760 × 10^−9^	0.0278
	Max	5.7937 × 10^−280^	4.5953 × 10^−85^	6.3352 × 10^−174^	8.3072 × 10^−8^	0.0119
	std	0	1.0275 × 10^−85^	0	1.9859 × 10^−8^	0.0559
F_5_	Avg	2.8139 × 10^−8^	5.3045	0.8858	4.2662	0.0093
	Min	2.3218 × 10^−12^	2.9575	0.3080	3.9462	93.3814
	Max	2.4718 × 10^−7^	8.9400	1.3964	4.6958	4.3471
	std	6.1393 × 10^−8^	1.8656	0.3123	0.1866	1.2890 × 10^3^

**Table 5:** Performance analysis of the proposed in multimodal benchmark functions.

Fun.	Index	İHBA	BKA	HBA	DO	GOOSE
F_6_	Avg	1.0477	2.2274	1.7344	0.9980	12.1950
	Min	0.9980	0.9980	0.9980	0.9980	1.9920
	Max	1.9920	10.763	10.7632	0.9980	23.8094
	std	0.2223	2.5403	2.2169	2.9263 × 10^−16^	6.9922
F_7_	Avg	0.3979	0.3979	0.3997	0.3979	0.3979
	Min	0.3979	0.3979	0.3979	0.3979	0.3979
	Max	0.3979	0.3979	0.4059	0.3979	0.3979
	std	0	0	0.0024	9.6669 × 10^−13^	2.3278 × 10^−8^
F_8_	Avg	0	0	0	0	2.4179 × 10^−7^
	Min	0	0	0	0	1.8959 × 10^−8^
	Max	0	0	0	0	1.1486 × 10^−6^
	std	0	0	0	0	2.9066 × 10^−7^
F_9_	Avg	0	0	0	0.0779	22.5705
	Min	0	0	0	2.5535 × 10^−15^	6.4134
	Max	0	0	0	0.3694	35.6370
	std	0	0	0	0.0889	8.6263
F_10_	Avg	4.4441 × 10^−16^	8.8818 × 10^−16^	8.8818 × 10^−16^	3.2859 × 10^−8^	7.1314
	Min	4.4441 × 10^−16^	8.8818 × 10^−16^	8.8818 × 10^−16^	9.4337 × 10^−9^	0.0062
	Max	4.4441 × 10^−16^	8.8818 × 10^−16^	8.8818 × 10^−16^	6.9182 × 10^−8^	18.7378
	std	0	0	0	1.7635 × 10^−8^	8.9577

**Table 6:** Description of the histopathological imaging dataset.

Dataset	Grade	Normal	OSCC	Total
100×	Training (%80)	71	351	422
	Testing (%20)	18	88	106
	Total	89	439	528
400×	Training (%80)	161	396	557
	Testing (%20)	40	99	139
	Total	201	495	696

**Table 7:** Statistical results for a 100× magnification factor of the dataset with 5 runs.

Method	Mean	Min	Max	Std
BKA	1.0341	1.0196	1.0612	0.0202
DO	1.0239	1.0000	1.0505	0.0196
Goose	1.0318	1.0196	1.0400	0.0111
HBA	1.0320	1.0097	1.0612	0.0198
iHBA	1.0239	1.0000	1.0505	0.0196

**Table 8:** Statistical results for a 400× magnification factor of dataset-1 with 5 runs.

Method	Mean	Min	Max	Std
BKA	1.0859	1.0775	1.0944	0.00599
DO	1.0816	1.0610	1.1393	0.03296
Goose	1.0762	1.0451	1.1031	0.02376
HBA	1.0681	1.0451	1.1031	0.02833
iHBA	1.0639	1.0296	1.1300	0.04112

**Table 9:** Performance comparison of metaheuristics-based feature selection methods for a 100× magnification factor of the dataset.

Method	Accuracy	Precision	Recall	F1-Score
HBA	0.9519	0.9595	0.9726	0.9660
BKA	0.9615	10.000	0.9429	0.9706
Goose	0.9712	0.9677	0.9836	0.9756
DO	0.9712	0.9841	0.9688	0.9764
iHBA	0.9808	1.000	0.9718	0.9857

**Table 10:** Classification results for different types of feature selection algorithms for a 100× magnification factor.

Method	Accuracy	Precision	Recall	F1-Score	Sel. Feats.
iHBA	0.9808	1.0000	0.9718	0.9857	1600
Relieff	0.9326	0.9220	0.9861	0.9326	1200
MRMR	0.9519	0.9466	0.9861	0.9519	20
NCA	0.9519	0.9859	0.9459	0.9655	63
Chi2 Test	0.9230	0.9682	0.9104	0.9384	100

**Table 11:** Classification results for different types of feature selection algorithms for a 400× magnification factor.

Method	Accuracy	Precision	Recall	F1-Score	Sel. Feats.
iHBA	0.9808	0.9855	0.9855	0.9855	1881
Relieff	0.8633	0.8772	0.8065	0.8403	1200
MRMR	0.8417	0.9000	0.7258	0.8036	20
NCA	0.8633	0.8909	0.7903	0.8376	2000
Chi2 Test	0.8273	0.8654	0.7258	0.7895	100

**Table 12:** Classification results of the proposed method with other optimizers for a 400× magnification factor.

Method	Accuracy	Precision	Recall	F1-Score	N-Feats
HBA	0.9423	1.000	0.9143	0.9552	1728
BKA	0.9712	0.9844	0.9692	0.9767	1523
Goose	0.9712	10.000	0.9589	0.9790	1609
DO	0.9519	0.9577	0.9714	0.9645	1537
iHBA	0.9808	0.9855	0.9855	0.9855	1881

**Table 13:** Performance comparison of the deep feature extraction networks with the proposed method.

Method	BCC (%)
ResNet18	95.45
ViT	89.39
ResTransNet	96.21
Proposed Method	98.08

## Data Availability

The datasets used in this study are publicly available and have been described in detail in the manuscript. These datasets can be accessed through their respective public repositories and sources cited in the paper.

## Abbreviations

The following abbreviations are used in this manuscript:

OSCC	Oral Squamous Cell Carcinoma
HBA	Honey Badger Algorithm
iHBA	Improved Honey Badger Algorithm
CLAHE	Contrast-Limited Adaptive Histogram Equalization
SVM	Support Vector Machine
FCM	Fuzzy C-Means
CNN	Convolutional Neural Network
ViT	Vision Transformer
SOA	Seagull Optimization Algorithm
PSO	Particle Swarm Optimization
BKA	Black-Winged Kite Algorithm
DO	Dandelion Optimization
Goose	Goose Algorithm
std	Standard Deviation
MRMR	Minimum Redundancy Maximum Error
NCA	Neighborhood Component Analysis
